# Using Benford’s law to assess the quality of COVID-19 register data in Brazil

**DOI:** 10.1093/pubmed/fdaa193

**Published:** 2020-10-24

**Authors:** Lucas Silva, Dalson Figueiredo Filho

**Affiliations:** Universidade Estadual de Ciências da Saúde do Estado de Alagoas, Maceió, Alagoas 57010-300, Brazil; Universidade Federal de Pernambuco, Recife, Pernambuco 50670-901, Brazil

**Keywords:** COVID-19, first digit law, Newcomb–Benford law, SARS-CoV-2

## Abstract

We employ Newcomb–Benford law (NBL) to evaluate the reliability of COVID-19 figures in Brazil. Using official data from February 25 to September 15, we apply a first digit test for a national aggregate dataset of total cases and cumulative deaths. We find strong evidence that Brazilian reports do not conform to the NBL theoretical expectations. These results are robust to different goodness of fit (chi-square, mean absolute deviation and distortion factor) and data sources (John Hopkins University and Our World in Data). Despite the growing appreciation for evidence-based-policymaking, which requires valid and reliable data, we show that the Brazilian epidemiological surveillance system fails to provide trustful data under the NBL assumption on the COVID-19 epidemic.

## Introduction

COVID-19, ongoing pandemic, infected almost 30 million people worldwide, and it is getting close to 1 million deaths as of September 2020.^1^ Unlike other epidemics events, SARS-CoV-2 infection is closely covered by media outlets, academics and national governments in real time. The amount of available data grows as fast as the epidemic itself, which presents a unique opportunity for policymakers to develop counteract measures. In any case, evidence-based interventions require reliable data.

In early June 2020, the Coronavirus panel, which is the primary official platform for monitoring COVID-19 data in Brazil, went offline due to political reasons. The national government suggested arbitrary changes on variable measurements and report standards that were against international epidemiological guidelines. After several days without updates, the system returned without estimates of incidence and mortality, in addition to displaying systematic inconsistencies such as negative cases and measurement error.^2^ The Brazilian data blackout got international visibility when John Hopkins University threatened to drop Brazil from the worldwide dataset. One week later, the Federal Supreme Court ruled that the Brazilian Ministry of Health must comply with the World Health Organization standards and disclose full data.

In this paper, we employ Newcomb–Benford law (NBL) to evaluate the reliability of COVID-19 data in Brazil. Using official aggregate information, we apply a first digit test for a national dataset of total cases and cumulative deaths.

## Data and methods

Benford’s law, initially proposed by Simon Newcomb, is an amusing example of a mathematical pattern that accurately describes a widely diverse collection of data.^3^ Based on frequency counts, NBL postulates that individual digits appear more frequently than others, where 1 is the most common first digit, leading nearly 30% of the time and 9 is the least common, with an expected frequency of less than 5%. Surprisingly, Benford’s law fits not only well-known distributions such as the power of 2 and Fibonacci sequence, but also it describes a sizeable broad dataset of natural numbers. In particular, NBL is widely applied as a fraud detection tool in different fields, from tax auditing, banking, quality of survey data, toxic emissions to election outcomes.

The following equation gives the probability of digit *d* occurring as the first significant digit:

$$P(d)={\mathit{\log}}_{10}\left(\frac{1+d}{d}\right)\ for\ d\in \left\{1,\dots, 9\right\}$$

As the NBL distribution of first digits follows an exponential distribution, it is particularly suited to examine the infectious disease patterns that exhibit increasing changes over time, especially in the early stages of the spread. Using official data from February 25 to September 15, we apply a first digit test for national aggregated records (cases and deaths). To estimate to which extent observed data conform to Benford’s law’s theoretical expectation, we used the chi-squared (χ2) test of the goodness of fit, which is the most common statistical procedure to assess the null hypothesis. We also examine the mean absolute deviation (MAD) and the distortion factor (DF) to get more robust results.

The data were analyzed using R Statistical 3.6.3, and all significance tests were two sided. To estimate NBL functions, we used the *benford.analysis* package developed by Cinelli.^4^ All data obtained were from Cota.^5^ Replication materials, including raw data and computational scripts, are available on https://osf.io/74xjc/.

## Results

To make comparisons more precise, we can compute the formal goodness-of-fit tests. The null hypothesis is that the observed data follow the NBL. Therefore, the smaller the *P* value, the higher our confidence in rejecting the null hypothesis that observed distribution conforms to the NBL theoretical expectations. As Table 1 shows, for both variables we analyze, the χ2 test leads one to reject the null hypothesis at conventional levels of significance.

**Table 1:** COVID-19 total cases and cumulative deaths in Brazil

*Digit*	*NBL*	*Covid-19 total cases*	*Covid-19 total deaths*
1	30.1	26.6	35.5
2	17.6	23.2	10.9
3	12.5	19.7	8.2
4	9.7	11.8	8.7
5	7.9	3.9	8.7
6	6.7	3.9	6.6
7	5.8	3.9	8.2
8	5.1	3.0	6.0
9	4.6	3.9	7.1
N		203	203
χ2		23.363	14.115
MAD		0.033	0.027
DF		−8.139	5.179
Mantissa		0.471	0.494

**Fig. 1:**
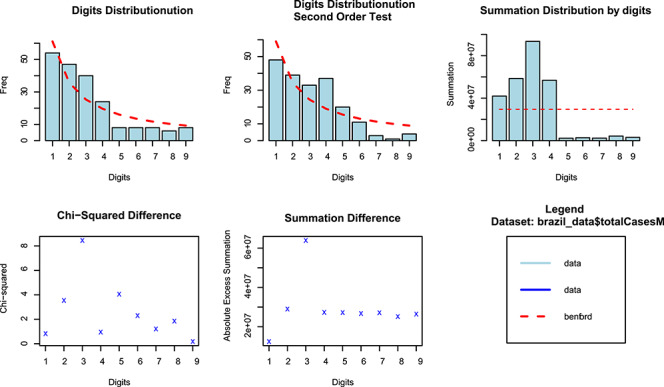
NBL estimates of COVID-19 total cases.

**Fig. 2:**
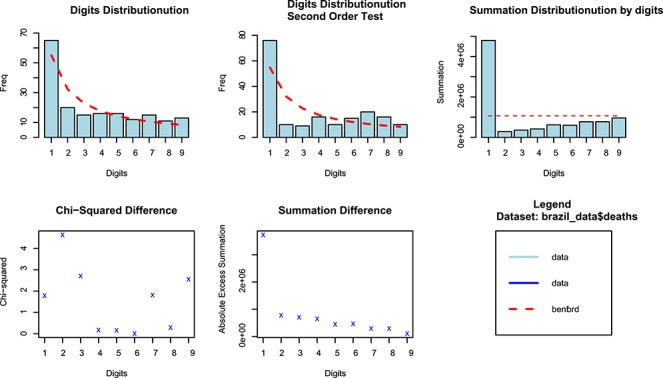
NBL estimates of COVID-19 total deaths.

Similar to *Z*-statistic, the χ2 test is highly sensitive to the sample size and tends to reject the null even for small departures from the expected distribution. The MAD test is more robust since it ignores the number of records. The higher the MAD, the larger the average difference between the observed and theoretical distributions. MAD values above 0.015 suggest nonconformity. Following this criterion, we should conclude that Brazilian data are highly discordant to the NBL distribution (see Figs 1 and 2).

Finally, the DF statistic exams the digit patterns to indicate whether the data appear to be underestimated or overestimated and the deformity’s magnitude. Table 1 shows that COVID-19 total cases are likely to be underestimated since reports exhibit an excess of lower digits. Under NBL, we expect that mantissa is uniformly distributed with an average of 0.5. Suppose figures are manipulated downward by exclusively altering the mantissa. In that case, the mean observed value will be less than the expected average, which seems to be the case of the COVID-19 Brazilian epidemiological surveillance system.

## Discussion

NBL is a well-established statistical tool for detecting suspicious activities in data. The evidence reported here does not constitute proof of illegality, but we have identified consistent departures of Brazilian COVID-19 reports from NBL. Our findings are robust to different empirical tests and data sources, which increases our confidence that the epidemiological surveillance system fails to provide trustful data on the severe acute respiratory syndrome coronavirus 2 (SARS-CoV-2) epidemics in Brazil.

Evidence-based policymaking requires valid and reliable information, which depends on institutional mechanisms of transparency and disclosure. Without open data, it is impossible to evaluate the extent to which government interventions affected the outcomes they were designed to change. We are aware of the technical obstacles of measuring a new and complex disease such as COVID-19. However, effective policy policies rely on data quality and availability. We hope to see more sophisticated practices of gathering records by the Brazilian Minister of Health to facilitate data sharing and a more careful analysis of these critical data.

## Funding

This work was financially supported by Funding agency as coordination for the improvement of Higher Education Personnel (CAPES).

## Conflicts of interest

There is no conflict of interest.

## Notes

**Lucas Silva**, Research Assistant

**Dalson Figueiredo Filho**, Professor
